# Evaluating the impact of pop-off mechanisms in boys with posterior urethral valves

**DOI:** 10.3389/fped.2022.1014422

**Published:** 2022-10-18

**Authors:** T. Delefortrie, C. Ferdynus, A. Paye-Jaouen, J. L. Michel, E. Dobremez, M. Peycelon, A. El Ghoneimi, L. Harper

**Affiliations:** ^1^ Department of Pediatric Surgery, Hôpital Robert Debré, Paris, France; ^2^ Department of Pediatric Surgery, CHU F Guyon, Saint-Denis de La Réunion, France; ^3^ Department of Pediatric Surgery, CHU Pellegrin-Enfants, Bordeaux, France; ^4^ Unité de Soutien Méthodologique, CHU F Guyon, Saint-Denis de La Réunion, France; ^5^ Hôpital Robert Debré, Centre de Référence des Malformations Rares des Voies Urinaires (MARVU), Paris, France

**Keywords:** posterior urethral valves, pop-off mechanism, renal function, urinoma, VURD, bladder diverticula

## Abstract

**Introduction:**

Posterior urethral valves are urethral leaflets that cause Lower Urinary Tract Obstruction (LUTO) in boys and are associated with congenital renal dysplasia and abnormal bladder function. They affect 1:4,000 to 1:25,000 births and can be responsible for End-Stage Renal Failure in childhood. There have been several studies on the effect of pop-off mechanisms in boys with posterior urethral valves, but results are contradictory. We aimed to assess and discuss the effect of pop-off mechanisms on renal function in a large cohort of patients.

**Patients and method:**

Boys with PUV with and without pop-off mechanisms (urinoma, VURD or giant bladder diverticula) were divided into three severity groups for renal function according to their nadir creatinine (low-risk NC < 35 μmol/L, intermediate-risk NC between 35 and 75 μmol/L, and high-risk NC > 75 μmol/L). We compared children with and children without pop-off mechanisms for mean renal function as well as patient distribution within each severity group.

**Results:**

We included 137 boys of which 39 had a pop-off mechanism. Patients had complete data for at least 5 years follow-up. Though there was no significant statistical difference in mean renal function between the pop-off and non-pop-off group, patient distribution within each severity group varied according to whether patients had a pop-off mechanism or not.

**Conclusion:**

Though there was no significant difference in mean renal function between boys with and without pop-off mechanisms, it is possible that these are two different patient populations and direct comparison is not possible.

## Introduction

Posterior urethral valves cause both bladder and renal damage because of increased intravesical pressure during fetal kidney development. Some cases of posterior urethral valves are associated with pressure “pop-offs” that, in theory, could buffer elevated intravesical pressures thus preserving the urinary tract (1). Several authors have studied the effect of pop-off mechanisms both on renal or bladder function with conflicting results (1–8). We aimed to assess the effect on renal function of the pop-off mechanism in a large cohort of patients.

## Patients and methods

In this retrospective study, we included all boys with prenatally diagnosed lower urinary tract obstruction born between 2000 and 2013 in two University Hospitals. Information came from two prospectively gathered databases. We excluded patients who presented a LUTO other than PUV, children who presented multiple birth defects and neonatal deaths. We did not exclude some patients who were subsequently lost to follow-up if sufficient relevant data was available, but those with insufficient data were excluded. We included data on gestational age at birth, age at surgery, presence or absence of a pop-off mechanism, nadir creatinine (NC), i.e., lowest creatinine during the first year of life, long-term renal function (defined by the Chronic Kidney Disease classification based on latest serum creatinine), presence of febrile urinary tract infections. Patients had complete data for at least 5 years follow-up.

Boys with PUV with and without pop-off mechanisms were divided into three severity groups for renal function according to their NC using the classification proposed by the team from Birmingham (7). This classification divides patients into three risk groups for renal failure: low-risk NC < 35 μmol/L, intermediate-risk NC between 35 and 75 μmol/L, and high-risk NC > 75 μmol/L. Patients were also classified according to their Chronic Kidney Disease stage as determined by their Glomerular Filtration Rate.

We defined pop-off mechanism as presence of either: VURD (Posterior urethral valve, Unilateral vesicoureteral reflux, Renal dysplasia) syndrome as defined by voiding cystogram and DMSA scan; urinoma or urinary ascites, confirmed by ultrasound, or a large bladder diverticula, defined as a diverticula measuring more than a third of the bladder volume.

Characteristics of patients were described as frequencies and proportions for categorical variables and mean with standard deviation or median and quartiles for quantitative variables. Bivariate analyses were performed using Pearson Chi-Square or Fisher exact test for qualitative data, and Student's *t*-test or Mann–Whitney test for quantitative data, as appropriate. All tests were performed at a 2-tailed type I error of 5%.

All analyses were performed using SAS 9.4 (SAS Institute Inc., Cary, NC, USA).

## Results

The original series included 172 patients. We excluded 4 other LUTOs, 2 neonatal deaths and 29 lost to follow-up or insufficient data. We therefore included 137 boys who had complete renal function evaluation, of which 39 (28.5%; IC95%: 20.9–36.0) had a pop-off mechanism. Median follow-up was 8.3 years [IQR: 6.9–12.6].

Characteristics of patients are summarized in Table 1. Comparisons of patients with and without pop-off syndrome are presented in Table 2. All children had bilateral hydronephrosis and large bladders on prenatal ultrasound. All had confirmed valves on postnatal VCUG. Presence and grade of associated VUR was equivalent in both groups.

**Table 1:** Patient characteristics. Data are presented as *n* (%) for qualitative variables and as mean (sd) or median (*p* 25 – *p* 75) for quantitative variables.

	All Cohort *n* = 137
**Gestational age at birth (weeks)**	37.7 (2.2)
**Age at valve resection (days)**	1.0 (0.0–5.0)
**Pop-off, *n* (%)**	39/137 (28.5)
VURD syndrome	19/137 (13.9)
Urinoma	16/137 (11.7)
Bladder diverticula	9/137 (6.6)
**Nadir creatinine (*μ*mol/l) (<1 year of age)**	42.0 (26.4)
NC < 35	57/137 (41.6)
35 ≤ NC ≤ 75	70/137 (51.1)
NC > 75	10/137 (7.3)
**CKD stage, *n* (%) (at latest follow-up)**	** **
1	54/101 (53.5)
2	27/101 (26.7)
3	12/101 (11.9)
4	2/101 (2.0)
5	6/101 (5.9)
**Chronic renal failure, *n* (%) (latest follow-up)**	48/102 (47.1)
**Febrile urinary tract infections, *n* (%)**	68/118 (57.6)

**Table 2:** Comparisons of patients with and without pop-off syndrome. Data are presented as *n* (%) for qualitative variables and as mean (sd) or median (*p* 25 – *p* 75) for quantitative variables.

	Pop-off syndrome *n* = 39	Non pop-off syndrome *n* = 98	*p*-value
**Gestational age at birth (weeks)**	36.9 (2.1)	38.1 (2.2)	0.02
**Age at valve resection (days)**	0.0 (0.0–4.0)	1.0 (0.0–6.0)	0.05
**Nadir Creatinine (μmol/l) (<1 year of life)**	35.7 (12.2)	44.5 (29.9)	0.31
NC < 35	17/39 (43.6)	40/98 (40.8)	0.11
35 ≤ NC ≤ 75	22/39 (56.4)	48/98 (49.0)	
NC > 75	0/39 (0.0)	10/98 (10.2)	
**CKD stage, *n* (%) (at latest follow-up)**			
1	18/32 (56.2)	36/69 (52.2)	0.28
2	11/32 (34.4)	16/69 (23.2)	
3	3/32 (9.4)	9/69 (13.0)	
4	0/32 (0.0)	2/69 (2.9)	
5	0/32 (0.0)	6/69 (8.7)	
**Chronic renal failure, *n* (%) (latest follow-up)**	14/32 (43.7)	34/70 (48.6)	0.65
**Febrile urinary tract infections, *n* (%)**	20/32 (62.5)	48/86 (55.8)	0.51

**Figure 1:**
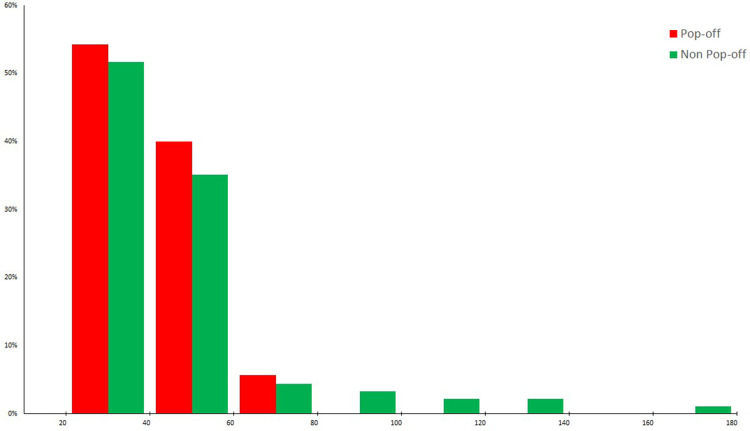
Patient distribution (%) according to nadir creatinine.

There was a non-significant difference in mean renal function, as determined by latest creatinine, between both the pop-off and non-pop-off group: respectively 35.7 +/−12.2 µmol/l and 44.5 +/−29.9 µmol/l (*p* = 0.31). However, patient distribution within each severity group (based on nadir creatinine) varied according to whether patients had a pop-off mechanism or not (Figure 1). Whilst in the non-pop-off group 10% of patients presented a nadir creatinine (NC) > 75 μmol/L, none of the pop-off patients presented NC > 75 μmol/L. The severity groups tallied well with long-term renal function as 20.0% of boys with a NC < 35 μmol/L developed chronic renal disease vs. 62.5% with an NC between 35 and 75 μmol/L and 100% of those with NC > 75 μmol/L. At latest follow-up, in the pop-off group 90.6% of patients’ present stage 1 or 2 CKD and only 9.4% present stage 3 CKD, whilst none present stage 4 and 5 CKD. At latest follow-up, in the non-pop-off group 8 patients presented stage 4 and 5 CKD of which 7 have had a renal transplant.

## Discussion

Pressure pop-off mechanisms, and their putative protective effect on renal function in boys with posterior urethral valves, were initially described by Rittenberg et al. (1). These pressure buffering mechanisms include the VURD syndrome (unilateral vesicoureteral reflux and renal dysplasia syndrome) described a few years earlier (8, 9), large congenital bladder diverticula (8) and urinary extravasation with or without urinary ascites (2). In their original publication Rittenberg et al. compared 20 boys with pop-off mechanisms to 51 boys without and found both a difference in mean serum creatinine and a difference in the range of severity in each group, with more severe cases in the non-pop-off group. Since then, there have been other published reports that challenge these results, concluding that renal function ultimately deteriorates in patients with VURD syndrome (4–6).

There are several possible explanations for these discrepancies. As in many cases, there is quite a large disparity in patients and methods between published articles rendering direct comparison and analysis difficult and meta-analysis impossible. There are for instance large variations in the proportion of children with pop-off mechanisms in the study groups, ranging from 6 to 71%, which indicates significant differences in patient population. The accepted incidence for pop-off mechanisms is 30% (1) as in our series which included all patients with PUV. Most studies also include early and late diagnosis patients, who are known to evolve differently (10). And there are variations in studied outcome, some focused on serum creatinine and others on CKD stage, as well as variation in duration of follow-up. There are also differences in inclusion criteria, some articles focusing on VURD or urinomas, some studies including late-diagnosis PUV (11, 12).

For this study we decided to evaluate a large homogeneous series of patients with posterior urethral valves. We selected only children with prenatal diagnosis of PUV and immediate postnatal management, in order to exclude late diagnosis PUV patients who, as mentioned previously, are different patients.

We performed multiple analyses, of mean results but also of patient distribution using nadir creatinine and CKD classification because a single analysis gives only a limited view. We decided to classify renal function in three categories as again a binary classification between normal and abnormal is too restrictive for this population for whom the range of impairment goes from normal to early-onset end-stage renal disease.

Nevertheless, direct comparison of means between patients with a pop-off mechanism and those without shows no statistical difference. Does this mean pop-off mechanisms have no protective effect? This is obviously one of the possible explanations but pop-off mechanisms occur by definition in boys with high-pressure. Urinomas, giant diverticula or high-grade reflux appear because of high-pressure in a closed circuit. In the recent study by d'Oro et al., patients with pop-offs had worse bladder dynamics initially, which to them suggests that pop-offs are a manifestation of more excessive pressure build-up prior to valve ablation (8). High pressure, especially during renal tract development is associated with a high-risk of renal damage, yet in our series few patients with a pop-off mechanism had severe renal impairment. We cannot certify that our study, as well as previous studies, do not fail by assuming that these are two comparable populations. It is possible that pop-off mechanisms occur in a specific high-risk group of patients, who do benefit from this protective mechanism. Direct comparison with boys without pop-off mechanisms induces selection bias, as the non-pop-off group comprises patients who presented little or no elevated intravesical pressure. In this case, a more pertinent comparison would be to compare boys with and without pop-off mechanisms in a population with similar prenatal intravesical pressures, but there is however, for the moment, no reliable way to measure prenatal intravesical pressure. Patient distribution within each severity group in the pop-off and non-pop-off patients are different in our series, and can be interpreted as showing a tendency for less severe outcomes in the pop-off group.

We have a fairly large series with significant follow-up, however we cannot certify that a difference in renal function would appear later. Indeed, in the series by Okutesh et al. (13), the mean time of renal survival was calculated as 7.8 years. But again, we believe we might be comparing the incomparable.

Also, all pop-off phenomena do not necessarily have similar effect on kidney function. It can be postulated that at least affected kidney has suffered in VURD syndrome and perhaps in urinoma but both kidneys may be preserved in patients with bladder diverticula. We do not however have sufficient patients to allow for a full subgroup analysis. In our series different types of pop-off mechanisms seemed to have similar effects.

We have the impression that boys with pop-off mechanisms will not by definition fare better than boys without pop-off mechanisms, but that they will do better than they would themselves have done without pop-off.

## Data Availability

The raw data supporting the conclusions of this article will be made available by the authors, without undue reservation.
